# Quasilinearization numerical technique for dual slip MHD Newtonian fluid flow with entropy generation in thermally dissipating flow above a thin needle

**DOI:** 10.1038/s41598-021-94312-3

**Published:** 2021-07-23

**Authors:** Sohaib Khan, Farhad Ali, Waqar A. Khan, Anees Imtiaz, Ilyas Khan, Thabet Abdeljawad

**Affiliations:** 1 Department of Mathematics, City University of Science and Information Technology, Peshawar, 25000 Pakistan; 2 Department of Mechanical Engineering, College of Engineering, Prince Mohammad Bin Fahd University, Al Khobar, 31952 Kingdom of Saudi Arabia; 3 Department of Mathematics, College of Science Al-Zulfi, Majmaah University, Al-Majmaah, 11952 Kingdom of Saudi Arabia; 4 Department of Mathematics and General Sciences, Prince Sultan University, Riyadh, Kingdom of Saudi Arabia; 5 Department of Medical Research, China Medical University, Taichung, 40402 Taiwan; 6 Department of Computer Science and Information Engineering, Asia University, Taichung, Taiwan

**Keywords:** Engineering, Mathematics and computing, Physics

## Abstract

In the present article, we investigate the dual slip effect namely the velocity slip and thermal slip conditions on MHD flow past a thin needle. The entropy generation for the incompressible fluids that’s water and acetone that flowing above the thin needle is discussed. The energy dissipating term and the magnetic effect is included in the axial direction. The leading partial differential equations are transformed to ODE by an appropriate similarity transformation and solved using a numerical technique that is the Quasilinearization method. The terms for the rate of entropy generation, the Bejan number, and the irreversibility distribution ratio are discussed. Each dimensionless number is shown with velocity slip and also with the magnetic parameter is presented in graphical form. In the result, we conclude that the entropy generation rate is increasing with the increase in thermal slip parameter also some increasing effect is found as the size of the needle increases

## Introduction

Lee^1^ was the first who introduced the momentum boundary layer flow for Newtonian fluid above a thin needle. The condition by which similarity solution can exist, that's are the needle should be designed like a paraboloid of revolution. Lee considers only needles whose widths are similar to those of the boundary layer or slighter. These flows include a wide range of applications in aerospace, the flow of torpedoes, boats, acetone craft, and much more in marine engineering. Some applications in industries are transportation, coating of wires, lubrication, geothermal power generation, and blood flow problem in biomedical.

In any physical process, the conversion of energy is a necessary step. In the first law of thermodynamics, we discussed quantity (amount) of energy, and the second law, refer us to the quality (superiority), and the degradation of energy. The tool by which we measure the quality of energy is entropy or irreversibility. According to energy laws, during energy conversion to some useful work, energy is lost, by which the product performance is quite a week. This degradation of energy is relative to the generation of entropy. The greater the irreversibility, the larger will be the generation of entropy. The heat transfer and fluid friction are responsible for entropy generation, and the water has greater entropy than acetone^2^.

Bejan^3^ concluded that generally, heats transfer is associated swith the entropy generation's irreversibility. In the fluid flow process, the author found that the viscous effects, mainly heat transfer, high velocities of fluid flow, and temperature gradient, are responsible for the entropy generation rate. The energy that is consumed in any work has direct proportionality with irreversibility. The irreversibility process can be reduced as required. Against this background, Bejan presented the entropy generation rate in terms of heat exchange; countercurrent gas to gas exchangers can be thoroughly condensed^4^. Heating and cooling processes in industrial are critical to holding the stability and control heat transfer capacity by using the nanofluid. It is concluded that the heat transfer rate of Ag-water nanofluid is higher than the Ag-Kerosene nanofluid^5–7^. To maintain the quality of energy or reduce entropy generation, it is necessary to examine the entropy generation's distribution rate within the fluid volume. This entropy generation in thermodynamic systems terminates the available work and thus decreases its productivity. This loss occurs in various technical areas such as cooling of electronic devices, nuclear reactors, energy storage systems^8^.

Chen et al*.* ^9^ examined the forced or mixed convection heat transfer from the non-isothermal thin needle where the pressure gradient is ignored. Grosan and Pop^10^ reviewed the boundary layer of forced convection flows above non-isothermal thin needles, and the needle sinks in nanofluids, a numerical technique is used for its solution. Here two types of nanoparticles are discussed that's are metallic or non-metallic, like Aluminum Oxide where water is considered as the base liquid. Soid et al.^11^ investigated the boundary layer flow above the thin needle that's the nanofluids.

The viscous dissipation^12^ manifests itself in a noticeable increase in the liquid temperature because of the change in the liquid's kinetic energy into thermal energy due to liquids friction in the flow. The irreversibility of the fluid flow in shear forces and heat transfer, where work is done on adjacent layers where slight heat is generated due to its viscosity, is known as viscous dissipation. In microchannel, this effect is existing if the ratio of length to width ratio is significantly huge. First of all, Gebhart^13^ investigated the consequence of viscous dissipation in natural convection fluid. Kumar studied the fluid flow and heat transfer properties of an electrically conducting Casson fluid as it passes through an exponentially stretching curved surface with a convective boundary condition^14^.He investigated the effect of Joule heating on MHD nonNewtonian fluid flow across a curved surface that is exponentially extending^15^. Kumar^16^ investigated MHD micropolar nanofluid flow through a stretching surface with non-uniform heat source/sink: thermophoresis and Brownian motion effects He testified a remarkable increase in temperature due to work against the viscous forces where the kinetic energy is continuously converted to internal energy and because of dissipation. Saritha et al.^17^ ensured the heat transfer investigation about the boundary layer flow having Ostwald–de Waele relationship with viscous dissipation effects. The importance of viscous dissipation is the slight increase in temperature are detected in polymer dispensation streams such as extrusion at high rates. In aerodynamics, the high-speed acetone craft increases skin temperature within the boundary layer^18–22^. All previous discussions for thin needle problems focused on heat transfer analysis with entropy generation, and also, some of the energy equations involve an energy dissipation term. Still, no care was taken to understand how the thin needle's entropy generation with dual slip condition occurs in a parallel motion. It is also good to note that no works have been found in the existing papers for studying the dual slip effect on the boundary layer over a thin needle.

The current study aims to examine the effects of dual slip condition with magnetohydrodynamic MHD flow, the velocity ratio, and the viscous dissipation parameters on the entropy generation rate above thin needle has been discussed for water and acetone. The governing equations are transformed to ODE by suitable similarity transformation. The obtained equations are solved by numerical technique through the Quasilinearization technique.

## Mathematical formulation

**Figure 1:**
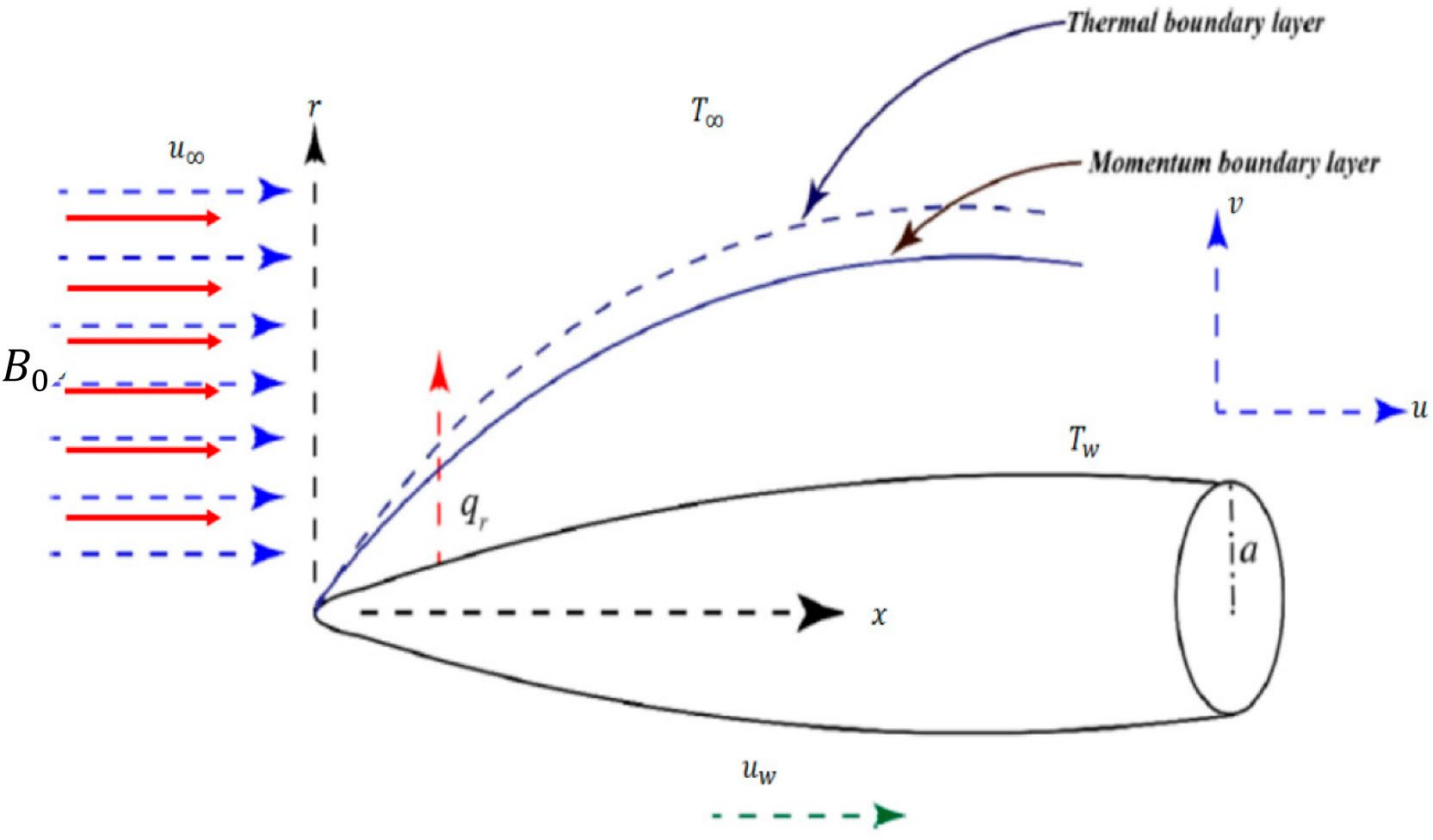
Schematic diagram for the given flow regime.

We consider an incompressible, steady, and laminar boundary layer flow in electrically conducting fluid above the thin needle that's moving at a constant velocity ${u}_{w}$. The thin needle is placed parallel to the free stream in the free stream as given in the given problem's geometry shown in Fig. 1. On the surface of the needle, the pressure gradient is negligible. The boundary of the thin needle is heated that ${T}_{w}>{T}_{\infty }$. Also, velocity slip and thermal slip conditions are taken into account at the wall, and the effect of the magnetic field is incorporated through the momentum equation^23^. Under the above assumptions, the governing equations, the governing continuity, momentum, and thermal energy equation can be written in cylindrical coordinates using the boundary layer assumption we get^4^.

1 $$ \frac{\partial }{\partial x}\left( {ru} \right) + \frac{\partial }{\partial r}\left( {rv} \right) = 0, $$

2 $$ u\frac{\partial u}{{\partial x}} + v\frac{\partial u}{{\partial r}} = \frac{\nu }{r}\frac{\partial }{\partial r}\left( {r\frac{\partial u}{{\partial r}}} \right) - \frac{{\sigma B_{0}^{2} u}}{\rho } $$

3 $$ u\frac{\partial T}{{\partial x}} + v\frac{\partial T}{{\partial r}} = \frac{\alpha }{r}\frac{\partial }{\partial r}\left( {r\frac{\partial T}{{\partial r}}} \right) + \frac{\nu }{{c_{p} }}\left( {\frac{\partial u}{{\partial r}}} \right)^{2} $$

where $u$ axial while $v$ is in the radial dimensions, $\alpha$ is thermal diffusivity, $\rho$ is density, $\nu$ shows kinematic viscosity, $\sigma$ is the electrical conductivity,${B}_{0}$ is the constant magnetic field strength, L is the velocity slip parameter, and D is the thermal slip coefficient^23^ for the given flow. The assumed boundary conditions are as follow.

4 $$ \left. \begin{gathered} u = u_{w} + L\left( {\frac{\partial u}{{\partial r}}} \right),\,\;v = 0,\,\,T = T_{w} + D\frac{\partial T}{{\partial r}} \,\,\,\,\,\,{\text{at }}r = R\left( x \right) \hfill \\ \,\,\,\,\,\,\,\,\,\,\,\,\,\,\,\,\,\,\,\,u \to u_{\infty } ,\,\,\,T \to T_{\infty ,} \,\,\,\,\,\,\,\,\,\,\,\,\,\,\,\,\,\,\,\,\,\,\,\,\,\,\,\,\,\,\,{\text{at }}r \to \infty \hfill \\ \end{gathered} \right\} $$

## Solution of the model

For the solution of momentum Eq. (2) and energy Eq. (3) of a thin needle, the similarity variables will be used, given by Ishak et al*.*^11^:

5 $$ \,\psi (x,\,r) = \nu x\,f(\eta )\quad \eta = \frac{{Ur^{2} }}{\nu x}, $$

where $\psi$ is the stream function we defined $u = \frac{1}{r}\frac{\partial \psi }{{\partial r}}$, and $v = - \frac{1}{r}\frac{\partial \psi }{{\partial x}}.$ The composite velocity is given by $U = u_{w} + u_{\infty } \ne 0$. Where $f()$ that setting $\eta = a$ (signifies the size of the thin needle). We have $R(x) = \left( {\frac{a\nu x}{U}} \right)^{1/2}$(propose the surface the size and shape of the needle). Encounter Eq. (5) in Eqs. (2) and (3) we get the following transform ODE with boundary condition.

6 $$ 2\eta f^{\prime\prime\prime} + f^{\prime\prime}\left( {2 + f} \right) - Mf^{\prime} = 0. $$

7 $$ f(a) = \frac{a\varepsilon }{2}, \, f^{\prime}(a) = \frac{\varepsilon }{2} + \beta f^{\prime\prime}(a), \, f^{\prime}(\infty ) \to \frac{1 - \varepsilon }{2}, $$

where $\varepsilon = \frac{{u_{w} }}{U} = \frac{{\text{needle velocity}}}{{\text{composite velocity}}}$ is called velocity ratio parameter, $M = \frac{{x\sigma B^{2} }}{U\rho }$ is MHD parameter, $\beta = \frac{L}{x}$ is the velocity slip coefficient.

### Skin friction

The expression for the dimensional skin friction is specified by^1^

8 $$ C_{f} = \left. {\frac{\mu }{{\rho \left( U \right)^{2} }}\left( {\frac{\partial u}{{\partial r}}} \right)} \right|_{r = R\left( x \right)} $$

After transformation using similarity variables, we get

9 $$ C_{f} \left( {{\text{Re}}_{x} } \right)^{1/2} = 8\sqrt a f^{\prime\prime}(a) $$

where ${\text{Re}}_{x} = \frac{Ux}{\nu }$ shows the local Reynolds number.

### Temperature distribution

For the dimensionless energy equation, the similarity variable is given by

10 $$ \,\theta \left( \eta \right) = \frac{{T - T_{\infty } }}{{T_{w} - T_{\infty } }}\quad \eta = \frac{{Ur^{2} }}{\nu x}, $$

So, the dimensionless energy equation, with the dimensionless boundary conditions, can be written as

11 $$ \eta \theta^{\prime\prime} + \left( {1 + 0.5\Pr f} \right)\theta^{\prime} + 4\eta Ec\Pr f^{{\prime\prime}{2}} = 0 $$

12 $$ \theta (a) = 1 + \gamma \theta^{\prime}(a), \, \theta {(}\infty {)} \to {0;}\,\,\, $$

where $\Pr = \frac{\nu }{\alpha },Ec = \frac{{U^{2} }}{{c_{p} T_{\infty } }},$ $\gamma = \frac{d}{r},$ shows the Prandtl, Eckert numbers, and $\gamma$ shows the initial value of thermal slip coefficient, respectively.

### Nusselt number

The dimensional Nusselt number presented in^2^

$$ {\text{Nu}}_{x} = \frac{ - x}{{T_{\infty } }}\left. {\frac{\partial T}{{\partial r}}} \right|_{r = R\left( x \right)} $$

After transformation using similarity variables, we get

13 $$ Nu_{x} \left( {{\text{Re}}_{x} } \right)^{ - 1/2} = - 2\sqrt a \theta^{\prime}(a) $$

## Entropy generation

The entropy generation rate per unit volume, in cylindrical coordinates, can be written as

14 $$ \dot{S}^{\prime\prime\prime}_{gen} = \frac{k}{{T_{\infty }^{2} }}\left[ {\left( {\frac{\partial T}{{\partial r}}} \right)^{2} + \left( {\frac{\partial T}{{\partial x}}} \right)^{2} } \right] + \frac{\mu }{{T_{\infty } }}\left\{ {2\left[ {\left( {\frac{\partial v}{{\partial r}}} \right)^{2} + \left( {\frac{\partial u}{{\partial x}}} \right)^{2} } \right] + \left( {\frac{\partial u}{{\partial r}} + \frac{\partial v}{{\partial x}}} \right)^{2} } \right\} + \frac{{\sigma B_{0}^{2} u^{2} }}{{T_{\infty } }}{. } $$

The finite and positives of the entropy production is because of the velocity and due to temperature gradient. Using the giving condition, we get Eq. (14) in term of the following form

15 $$ \dot{S}^{\prime\prime\prime}_{gen} = \frac{k}{{T_{\infty }^{2} }}\left( {\frac{\partial T}{{\partial r}}} \right)^{2} + \frac{\mu }{{T_{\infty } }}\left( {\frac{\partial u}{{\partial r}}} \right)^{2} + \frac{{\sigma B_{0}^{2} u^{2} }}{{T_{\infty } }}. $$

After transformation Eq. (15) become

16 $$ Ns = \frac{{\dot{S}^{\prime\prime\prime}_{prod} }}{{k\Omega^{2}_{T} \eta {\text{Re}} x/x^{2} }} = \theta^{{\prime}{2}} + 4Brf^{{\prime\prime}{2}} = N_{\Delta T} + N_{Fric} $$

$$ N_{\Delta T} = \theta^{{\prime}{2}} \, \left( {\text{Conductive irreversibility}} \right), $$

$N_{Fric} = 4\Omega \Pr Ecf^{{\prime\prime}{2}} ,$ (Irreversibility due to fluid friction)

$N_{M} = \Omega \Pr EcMf^{{\prime}{2}}$ (Irreversibility due to MHD)

$\Omega_{T} = \frac{{T_{w} - T_{\infty } }}{{T_{\infty } }}$ (Dimensionless temperature parameter).

Bejan^3^ analyzed the convective heat transfer in term of distribution ratio of irreversibility as follows

18 $$ \Phi = \frac{{\dot{S}^{\prime\prime\prime}_{prod,Fric} }}{{\dot{S}^{\prime\prime\prime}_{prod,\Delta T} }}\quad \Phi = \frac{{4Brf^{{\prime\prime}{2}} }}{{\theta^{{\prime}{2}} }} $$

In general, when we have the result, $\Phi > 1$ its mean irreversibility is significant due to fluid friction. When we have $0 < \Phi < 1$ the heat transfer is responsible for irreversibility. When Φ = 1, the irreversibility is due to both heat and fluid friction.

### Bejan number

The ratio of irreversibility is known by Bejan number^24^

19 $$ Be = \frac{{\dot{S}^{\prime\prime\prime}_{prod,\Delta T} }}{{\dot{S}^{\prime\prime\prime}_{prod} }} = \frac{{\frac{k}{{T_{\infty }^{2} }}\left( {\frac{\partial T}{{\partial r}}} \right)^{2} }}{{\frac{k}{{T_{\infty }^{2} }}\left( {\frac{\partial T}{{\partial r}}} \right)^{2} + \frac{\mu }{{T_{\infty } }}\left( {\frac{\partial u}{{\partial r}}} \right)^{2} + \frac{{\sigma B_{0}^{2} u^{2} }}{{T_{\infty } }}}}. $$

After similarity transformation, we get.

20 $$ Be = \frac{{\theta^{{\prime}{2}} }}{{\eta \theta^{{\prime}{2}} + \Omega^{2} \Pr Ec(4\eta f^{{\prime\prime}{2}} + Mf^{{\prime}{2}} )}} $$

$$ Be = \frac{{\theta^{{\prime}{2}} }}{{\eta \theta^{{\prime}{2}} + \Omega^{2} Br(4\eta f^{{\prime\prime}{2}} + Mf^{{\prime}{2}} )}} $$

$$ Br = \frac{Ec\Pr }{{\Omega_{T} }}\,\,(Brinkman\,number) $$

$Be = 1$ It shows heat transfer is responsible for the irreversibility.

$Be = 0$ It shows fluid friction is responsible for the irreversibility. Now if $Be > > \frac{1}{2}$, then heat transfer will be leading while,

$Be < < \frac{1}{2}$ Then irreversibility leads to fluid friction.

## Numerical method

A numerical technique of implicit finite difference scheme uses for nonlinear ordinary differential Eqs. (6) and (11) along with boundary conditions (7) and (12) are solved with the Quasi-linearization technique^25,26^. Replacing Eqns. (6) and (11) by

21 $$ A_{1}^{i} f_{\eta \eta \eta }^{i + 1} + A_{2}^{i} f_{\eta \eta }^{i + 1} + A_{3}^{i} f_{\eta }^{i + 1} = 0 $$

22 $$ A_{4}^{i} \theta_{\eta \eta }^{i + 1} + A_{5}^{i} \theta_{\eta }^{i + 1} + A_{6}^{i} f_{\eta \eta }^{2(i + 1)} = 0 $$

With following conditions

23 $$ \begin{gathered} \, \left. \begin{gathered} f^{i + 1} = \frac{a\varepsilon }{2}, \, f_{\eta }^{i + 1} = \frac{\varepsilon }{2} + A_{7}^{i} f_{\eta \eta } (a),\,\,\,\,\,\,\theta^{i + 1} { = }A_{8}^{i} \theta_{\eta }^{{\text{i + 1}}} {\text{ + 1 at }}\eta { = }a \hfill \\ \,\,\,\,\,\,\,\,\,\,\,\,\,\,\,\,\,\,\,\,\,\,\,\,\,\,f_{\eta }^{i + 1} = \frac{1 - \varepsilon }{2}{, }\theta^{i + 1} = 0 \, \,\,\,\,\,{\text{at }}\eta { = }\infty \hfill \\ \end{gathered} \right\} \hfill \\ \,\,\,\,\,\,\,\,\,\,\,\,\,\,\,\,\,\,\,\,\,\,\,\,\,\,\,\,\, \hfill \\ \end{gathered} $$

where the coefficients are given by:

24 $$ \begin{gathered} A_{1}^{i} = 2\eta \, A_{2}^{i} = 2 + f \hfill \\ A_{3}^{i} = - M\,\,\,\,\,\,\,\,\,\,\,\,\,\,\,\,\,\,\,\,\,\,\,A_{4}^{i} = \eta \hfill \\ A_{5}^{i} = \left( {1 + 0.5\Pr f} \right)\,\,\,\,\,A_{6}^{i} = 4\eta Ec\Pr \hfill \\ A_{7}^{i} = \beta \,\,\,\,\,\,\,\,\,\,\,\,\,\,\,\,\,\,\,\,\,\,\,\,\,\,\,\,\,A_{8}^{i} = \gamma \hfill \\ \end{gathered} $$

In Eqs. (21)–(24), the coefficient function of the *i*^th^ iterations are known, and functions of (*i* + 1)th iterations are unknown, and we need to determine with the help of boundary conditions. A second-order central difference formula was used to discretize Eqs. (22) and (23) in the transverse direction. After each iteration, the system of a linear algebraic equation is reduced. These equations are solved using MAPLE 2020. The convergence criterion between the two iterations is assumed to be 10^–5^.

## Results and discussion

The entropy generation $\dot{S}^{\prime\prime\prime}_{gen}$ for the steady incompressible flow of water and acetone is investigated above a tinny needle that's moving in the parallel stream. The effects of governing parameters on the Nusselt number $\mathrm{Nu}$, the rate of entropy generation $\dot{S}^{\prime\prime\prime}_{gen}$, and Bejan number $\mathrm{Be}$ are examined graphically. The problem is solved by a numerical technique using *dsolve* command in MAPLE software.

**Figure 2:**
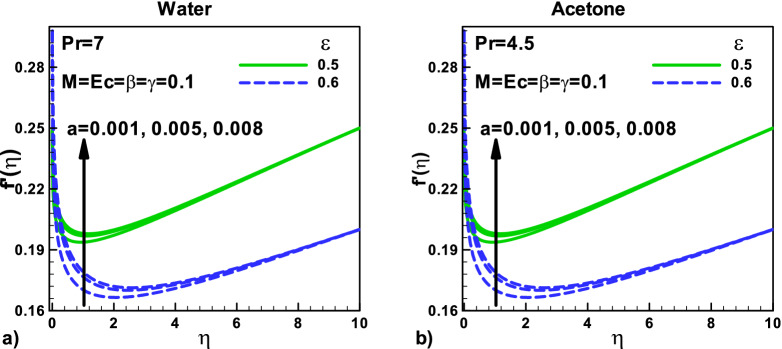
Effects of velocity ratio parameter on dimensionless velocity at different positions for (**a**) water (**b**) Acetone.

The effects of the velocity ratio parameter on the dimensionless velocity at different positions are displayed in Fig. 2a,b for the water and acetone, respectively. The thermal slip parameter γ has no effects on the dimensionless velocity in both cases because the momentum equation Eq. (6) is independent of this parameter. Also, the momentum equation is independent of the fluid properties. The thin needle's velocity is increased with the increase in the size of the needle, as shown in Fig. 2. For different velocities ratio ($\varepsilon = 0.5$) and ($\varepsilon = 0.6$) and different needle sizes, the dimensionless velocity increases for both water and acetone.

**Figure 3:**
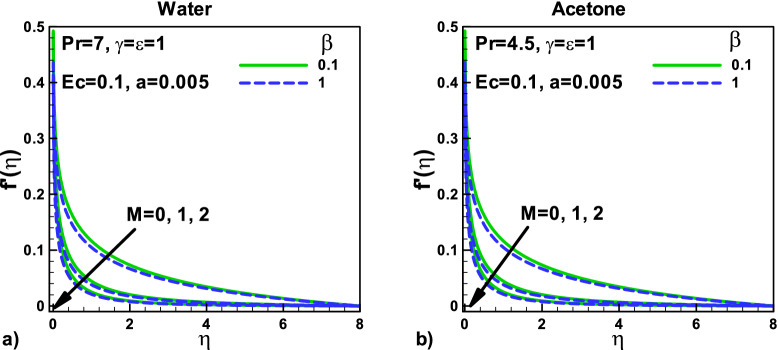
Effects of velocity slip and magnetic parameters on dimensionless velocity for (**a**) water (**b**) Acetone.

In Fig. 3, the effects of velocity slip and magnetic parameters on dimensionless velocity for (a) water and (b) Acetone, respectively. The thermal slip parameter γ has no effects on the dimensionless velocity in both cases, as the momentum equation Eq. (6) is independent of this parameter. The dimensionless velocities for both water and acetone are decreased due to an increase in the applied magnetic effect. The thermal slip parameter, velocity ratio parameter, and needle size are kept the same for both water and acetone.

**Figure 4:**
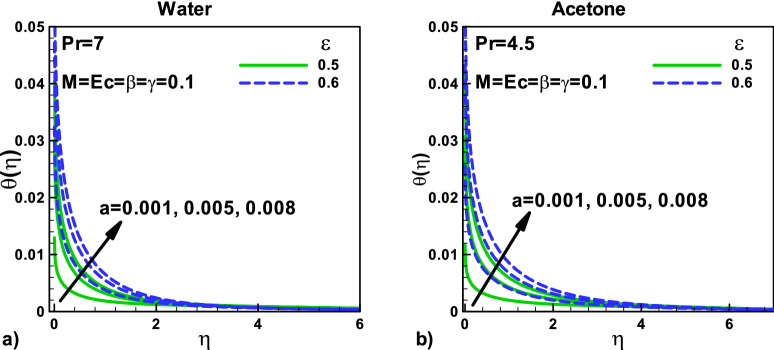
Effects of velocity ratio parameter on dimensionless temperature at different positions for (**a**) water (**b**) Acetone.

In Fig. 4, the velocity ratio parameter's effects on the dimensionless temperature at different positions for (a) water and (b) Acetone are shown**,** respectively. In both cases, the dimensionless temperature increase with the increase in the needle size. The magnetic effect, velocity slip, and thermal slip parameters are kept constant. When the tinny needle moves with the free stream velocity, the thermal resistance is higher, and as a result, the heat transfer rate is lower. The thermal slip parameter γ helps in increasing the dimensionless temperature inside the thermal boundary layer in both cases.

**Figure 5:**
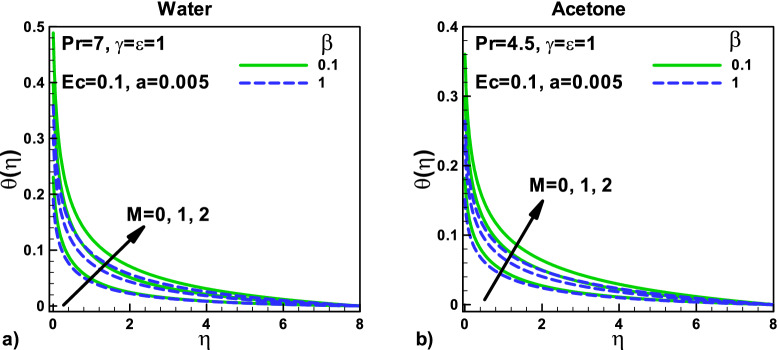
Effects of velocity slip and magnetic parameters on dimensionless temperature for (**a**) water (**b**) Acetone.

Figure 5: The effects of velocity slip and magnetic parameters on dimensionless temperature for (a) water and (b) acetone are shown, respectively. In both cases, the dimensionless temperature increases with the increase of magnetic parameters for different velocity slip parameters. When the tinny needle moves with the free stream velocity, the thermal resistance is higher, and as a result, the heat transfer rate is lower. The thermal slip parameter γ helps increase the dimensionless temperature inside the thermal boundary layer in both cases. The comparison shows the higher surface temperature for water under the same conditions. Due to the lower Prandtl number ${\varvec{P}}{\varvec{r}}$, the thermal boundary layer thickness for water is higher than acetone.

**Figure 6:**
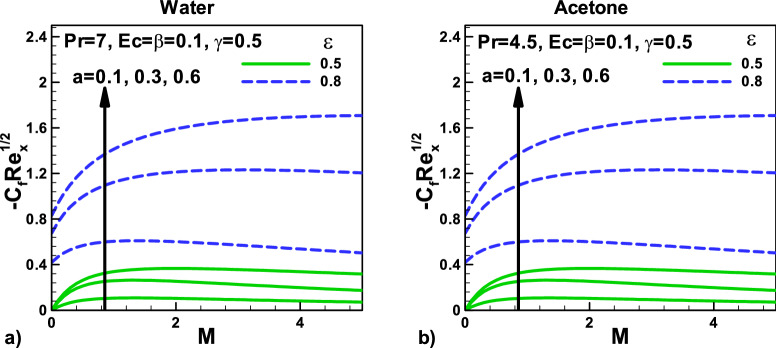
Variation of skin friction with magnetic field and velocity ratio parameter at different positions for (**a**) water (**b**) Acetone.

Figure 6: Variation of skin friction with magnetic field and velocity ratio parameter at different positions for (a) water and (b) Acetone. The velocity ratio parameter $\varepsilon = 0.5$ and $\varepsilon = 0.8$ are shown. Note for the Skin friction ${\mathbf{C}}_{{\varvec{f}}}$, that it is independent for thermal slip parameter γ and the fluid properties because there is no change for the different ${\varvec{P}}{\varvec{r}}$ and same velocity slip and thermal slip parameter. As the velocity ratio parameter $\varepsilon$ increases, the skin friction ${\mathbf{C}}_{{\varvec{f}}}$ increase due to increase in the free stream velocity and increase in magnetic effect.

**Figure 7:**
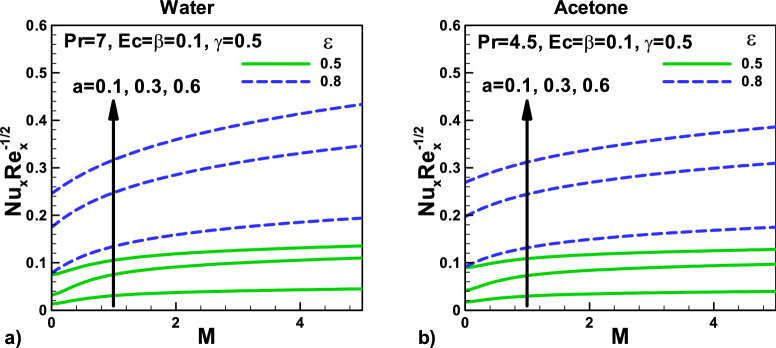
Variation of Nusselt number with magnetic field and velocity ratio parameter at different positions for (**a**) water (**b**) Acetone.

Figure 7: Variation of Nusselt number with magnetic field and velocity ratio parameter at different positions for (a) water and (b) Acetone**,** respectively. The three different needle sizes are selected to investigate these effects. As expected, the Nusselt number ${\varvec{N}}{\varvec{u}}$ have a direct relationship with the needle size as needle size increase it will enhance the Nusselt number ${\varvec{N}}{\varvec{u}}$ due to an increase in surface area in both cases. The thermal slip parameter γ increases with the temperature gradient, and as a result, the Nusselt number ${\varvec{N}}{\varvec{u}}$ increases. The Prandtl number ${\varvec{P}}{\varvec{r}}$ compares hydrodynamic and thermal boundary layer thickness and provides information about the heat transfer rate. Due to this reason, the Nusselt number ${\varvec{N}}{\varvec{u}}$ is higher for water, see Fig. 6a. An increase in the velocity ratio is due to the rise in the needle velocity, enhancing the heat transfer rate. This is true for both water and acetone.

**Figure 8:**
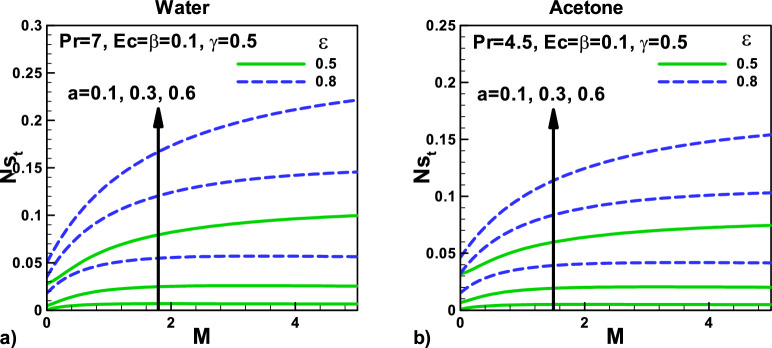
Variation of total entropy generation rate with magnetic field and velocity ratio parameter at different positions for (**a**) water (**b**) Acetone.

Figure 8: Variation of total entropy generation rate with magnetic field and velocity ratio parameter at different positions for (a) water and (b) acetone. The total entropy generation rate *N*_*st*_ includes the entropy generation due to heat and fluid friction and magnetic effect M, as shown in Eq. (15). *N*_*st*_ increases with the size of the needle. Both thermal slip and velocity slip effects are also displayed. However, *N*_*st*_ increases with increasing the needle's size due to an increase in the conduction of irreversibility. Due to higher Prandtl number ${\varvec{P}}{\varvec{r}}$, water shows higher heat transfer rates, and as a result, the entropy generation rates *N*_*st*_ are found to be higher for water. As the needle velocity increases, the heat transfer rate increases, increasing the entropy generation rate *N*_*st*_.

**Figure 9:**
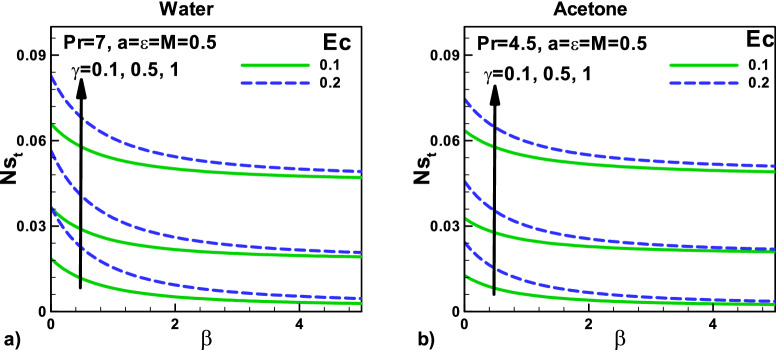
Variation of total entropy generation rate with velocity and thermal slip parameters for (**a**) water (**b**) Acetone.

In Fig. 9, the variation of total entropy generation rate with velocity and thermal slip parameters for (a) water and (b) acetone. The effects of Eckert number $Ec$ and the thermal slip parameter on the total entropy generation rate *N*_*st*_ are displayed in Fig. 8a,b for water and acetone, respectively. Eckert number $Ec$ also produces heating effects that increase the temperature gradients and increase the entropy generation rates *N*_*st*_.

**Figure 10:**
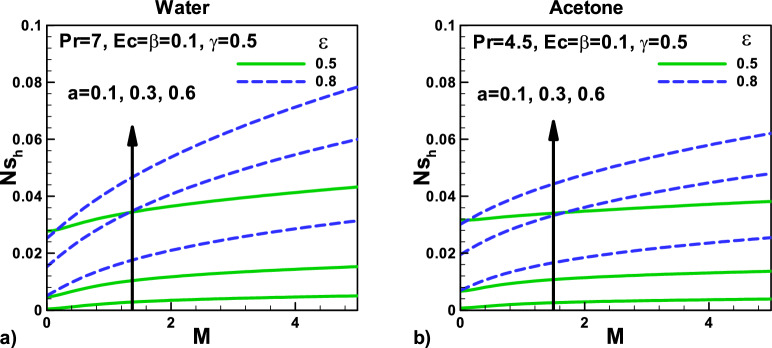
Variation of entropy generation rate due to heat with magnetic field and velocity ratio parameter at different positions for (**a**) water (**b**) acetone.

In Fig. 10 variation of entropy generation rate due to heat with magnetic field and velocity ratio parameter at different positions for (a) water and (b) Acetone. The total dimensionless entropy generation rate $\dot{S}^{\prime\prime\prime}_{gen}$ includes the entropy generation due to heat and fluid friction, as shown in Eq. (15). However, *N*_*sh*_ increases with increasing the needle's size due to an increase in the conduction irreversibility. Due to higher Prandtl number $Pr$, water shows higher heat transfer rates, and as a result, the entropy generation rates *N*_*sh*_ are found to be higher for water. As the needle velocity increases, the heat transfer rate increases, which helps in increasing the entropy generation rate *N*_*sh*_ due to the heating effect.

**Figure 11:**
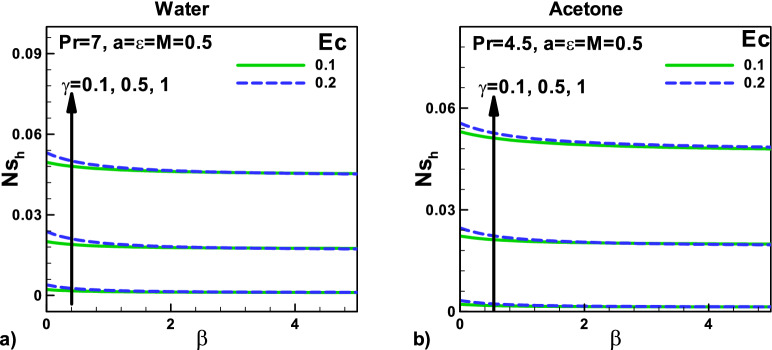
Variation of entropy generation rate due to heat with velocity and thermal slip parameters for (**a**) water (**b**) Acetone.

In Fig. 11, variation of entropy generation rate due to heat with velocity ${\varvec{\beta}}$ and thermal slip parameters ${\varvec{\gamma}}$ for (a) water and (b) Acetone. The effects of an increase in Eckert number ${\varvec{E}}{\varvec{c}}$ and the thermal slip parameter on the total entropy generation rate *N*_*sh*_ are also increasing, as displayed in Fig. 10 for water and acetone, respectively. Eckert number ${\varvec{E}}{\varvec{c}}$ also produces heating effects that increase the temperature gradients and increase the entropy generation rates *N*_*sh*_.

**Figure 12:**
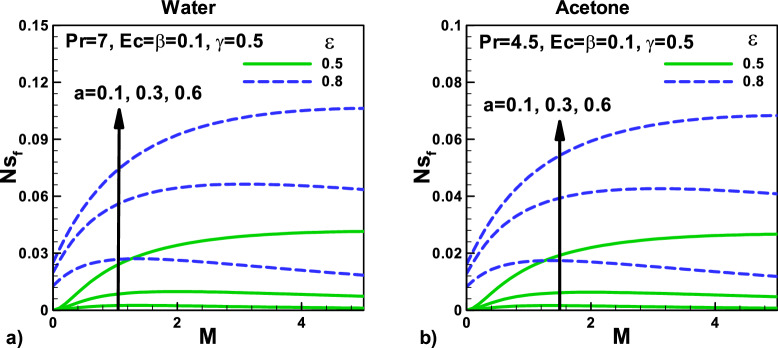
Variation of entropy generation rate due to friction with magnetic field and velocity ratio parameter at different positions for (**a**) water (**b**) Acetone.

Figure 12: The variation of entropy generation rate due to fluid friction with magnetic parameter ${\varvec{M}}$ and needle size ${\varvec{a}}$ for (a) water (b) acetone. The effect of variation in composite velocity and the increase in the size of the needle will increase entropy generation due to fluid friction. The impact of Eckert number ${\varvec{E}}{\varvec{c}}$ on the total entropy generation rate *N*_*sf*_ is also displayed with thermal slip parameter and with velocity slip ${\varvec{\beta}}$, in Figs. (a) and (b) for water and acetone, respectively.

**Figure 13:**
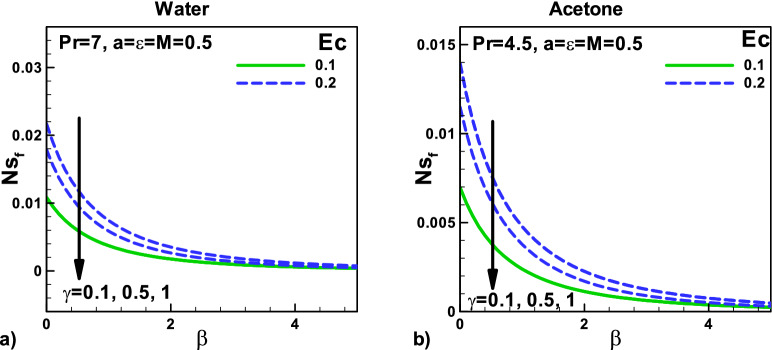
Variation of entropy generation rate due to friction with velocity and thermal slip parameters for (**a**) water (**b**) Acetone.

Figure 13: Variation of entropy generation rate due to velocity slip ${\varvec{\beta}}$ and thermal slip parameters for (a) water and (b) acetone. The effect of the magnetic parameter with composite velocity for the same needle involved. The impact of Eckert number ${\varvec{E}}{\varvec{c}}$ on the entropy generation rate *N*_*sf*_ due to fluid fraction with velocity slip is displayed in Fig. 12a,b for water and acetone, respectively. Eckert number ${\varvec{E}}{\varvec{c}}$ also produces heating effects that increase the temperature gradients and decrease the entropy generation rates due to fluid fraction *N*_*sf*_.

**Figure 14:**
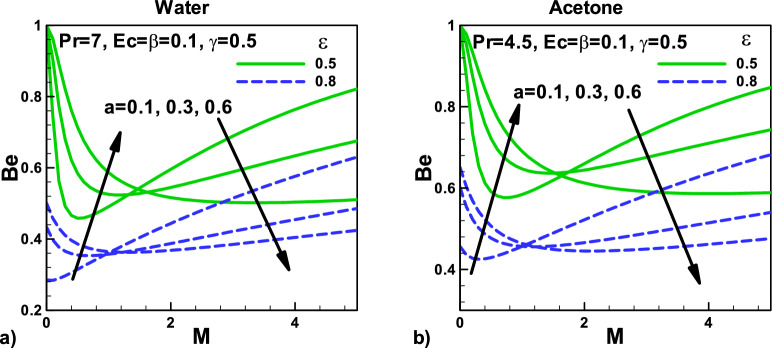
Variation of Bejan number with magnetic field and velocity ratio parameter at different positions for (**a**) water (**b**) Acetone.

Figure 14: Variation of Bejan number with magnetic field and velocity ratio parameter at different positions for (a) water and (b) acetone. The variation of Bejan number ${\varvec{B}}{\varvec{e}}$ with relevant parameters for acetone and water is depicted in Figs. 13 and 14, respectively. It shows the relative dominance of irreversibility due to heat transfer and fluid friction. For $0 \le Be < 0.5$, the irreversibility due to fluid friction dominates, and for $0.5 < Be \le 1$, the irreversibility due to heat transfer dominates. In Fig. 13a,b, the Bejan number *Be* is plotted for different needle sizes using acetone and water as working fluids. *Be* varies between 0.1 and 1 that shows the dominance of irreversibility due to fluid friction for smaller values of γ and the dominance of irreversibility due to heat transfer for larger values of γ. When the needle velocity increases, the irreversibility's due to heat transfer decrease. As a result, the *Be* also increases when magnetic parameter ${\varvec{M}}$ is between 0 and 1.And Be decrease for ${\varvec{M}}$ greater than 1. The viscous dissipation also helps in increasing the temperature, which increases the irreversibility due to heat transfer. Consequently, the Bejan number *Be* also increases Fig. 14a,b. Due to the higher Prandtl number ${\varvec{P}}{\varvec{r}}$ for water, the dimensionless surface temperature gradients, and higher Nusselt number ${\varvec{N}}{\varvec{u}}$, the irreversibility due to heat transfer is lower.

**Figure 15:**
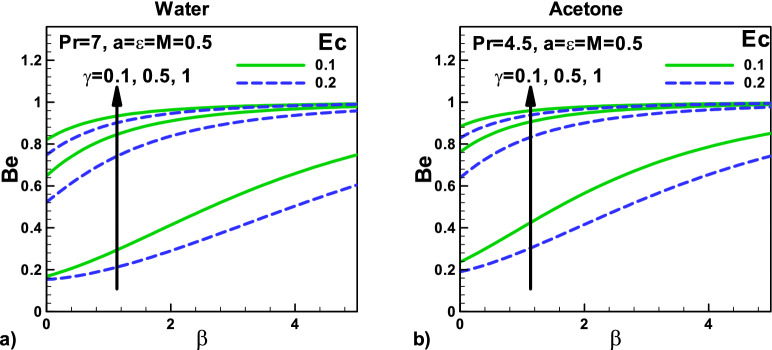
Variation of Bejan number with velocity and thermal slip parameters for (**a**) water (**b**) Acetone.

Figure 15: Variation of Bejan number with velocity ${\varvec{\beta}}$ and thermal slip parameters γ for (a) water (b) Acetone the variation of Bejan number ${\varvec{B}}{\varvec{e}}$ with relevant parameters for acetone and water is depicted in Figs. 13 and 14, respectively. It shows the relative dominance of irreversibility due to heat transfer and fluid friction. For $0 \le Be < 0.5$, the irreversibility due to fluid friction dominates, and for $\,\,0.5 < Be \le 1\,\,$, the irreversibility due to heat transfer dominates. In Fig. 15a,b, the Bejan number *Be* is plotted for different needle sizes using acetone and water as working fluids. It is evident that *Be* varies between 0.1 and 0.9 that shows the dominance of irreversibility due to fluid friction for smaller values of γ and the dominance of irreversibility due to heat transfer for larger values of γ. The viscous dissipation also helps in increasing the temperature, which increases the irreversibility due to heat transfer. Consequently, the Bejan number *Be* also increases Fig. 15a,b. Due to the higher Prandtl number ${\varvec{P}}{\varvec{r}}$ for water, the dimensionless surface temperature gradients, and hence higher Nusselt number ${\varvec{N}}{\varvec{u}}$, the irreversibility due to heat transfer is lower.

**Figure 16:**
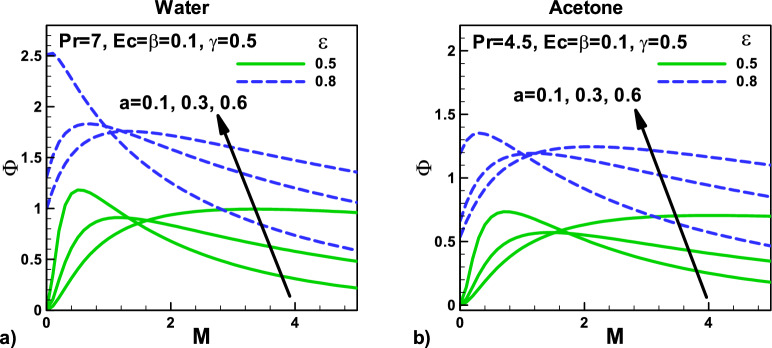
Variation of Irreversibility ratio with magnetic field and velocity ratio parameter at different positions for (**a**) water (**b**) Acetone.

Figure 16: Variation of Irreversibility ratio with magnetic field and velocity ratio parameter at different positions for (a) water (b) Acetone. The velocity ratio $\varepsilon$ and viscous dissipation parameters is demonstrated in Figs. 15 and 16 for both fluids. Different needle sizes and acetone and water are selected for this purpose. It is observed that as the needle size and the magnetic parameter M increase, the irreversibility distribution ratio $\Phi$ increase due to an increase in the heat transfer irreversibility, see Fig. 16a,b. The irreversibility distribution ratio $\Phi$ measures the influence of fluid friction irreversibility concerning thermal irreversibility. It reveals the dominance of fluid friction and heat transfer irreversibility in convection problems. It is noticed that as the needle velocity increases, the irreversibility distribution ratio $\Phi$ increases due to an increase in the fluid friction irreversibility in both cases. On the other side, viscous dissipation reduces the irreversibility distribution ratio $\Phi$ due to the dominance of heat transfer irreversibility in both cases, see Fig. 16a,b.

**Figure 17:**
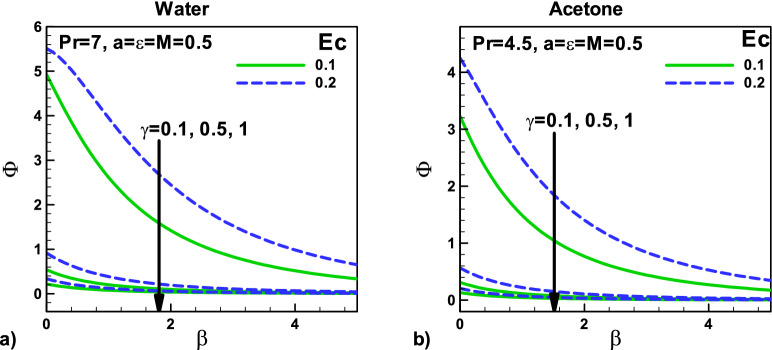
Variation of irreversibility ratio with velocity and thermal slip parameters for (**a**) water (**b**) Acetone.

Figure 17: Variation of irreversibility ratio with velocity $\beta$ and thermal slip parameters $\gamma$ for (a) water (b) Acetone, velocity ratio, MHD effect with an increase in Ec number is discussed for acetone and water are selected for this purpose. It is observed that as the needle size and the thermal parameter γ increase, the irreversibility distribution ratio $\Phi$ decreases due to an increase in the heat transfer irreversibility, see Fig. 17a,b. The irreversibility distribution ratio $\Phi$ measures the influence of fluid friction irreversibility with reference to thermal irreversibility. It reveals the dominance of fluid friction and heat transfer irreversibility in convection problems. It is noticed that as the thermal slip parameter increase, the needle velocity increases, whereas the irreversibility distribution ratio $\Phi$ decrease due to an increase in the fluid friction irreversibility in both cases. On the other side, viscous dissipation reduces the irreversibility distribution ratio $\Phi$ due to the dominance of heat transfer irreversibility in both cases.

## Conclusions

This work determines the effects of dual slip parameters $\beta$ and $\gamma$, velocity ratio $\varepsilon$, and viscous dissipation on the dimensionless parameters, including dimensionless velocity and temperature, skin fraction ${\mathrm{C}}_{f}$, Nusselt number $Nu$, the rate of entropy generation $\dot{S}^{\prime\prime\prime}_{gen}$, Bejan number, and irreversibility distribution ratio for acetone and water.

The following conclusions are derived from this investigation:

- The dual slip parameters $\beta$ and γ are the same for water and acetone.
- The magnetic parameter decreases the velocity of both water and acetone.
- As the needle's size increases, the dimensionless temperature increases with the magnetic effect and Ec number because Ec produces an increase in temperature.
- As the size of the needle increases, the skin friction increase with MHD.
- The needle size increases the Nusselt number $Nu,$ which is higher for water.
- Both the magnetic effect and velocity slip increase the total entropy generation *N*_*st*_.
- The entropy generation due to heat increases with magnetic and thermal slip parameters for both water and acetone.
- The entropy generation due to fluid friction is decreased due to an increase in the thermal slip parameter.
- The Bejan number shows dual nature. It increases when the magnetic effect is lower than 1 but $Be$ decrease for higher Magnetic parameter.
- The needle size and the magnetic parameter $M$ increase the irreversibility distribution ratio $\Phi$.
- The increase in dual slip velocity slip and thermal slip result decreases in the irreversibility distribution ratio $\Phi$.
